# Cultivation modes affect the morphology, biochemical composition, and antioxidant and anti-inflammatory properties of the green microalga *Neochloris oleoabundans*

**DOI:** 10.1007/s00709-024-01958-7

**Published:** 2024-06-12

**Authors:** C. Baldisserotto, S. Gessi, E. Ferraretto, S. Merighi, L. Ardondi, P. Giacò, L. Ferroni, M. Nigro, A. Travagli, S. Pancaldi

**Affiliations:** 1 Department of Environmental and Prevention Sciences, University of Ferrara, C.So Ercole I d’Este, 32, 44121 Ferrara, Italy; 2 Department of Translational Medicine, University of Ferrara, Via Fossato Di Mortara, 17-19, 44121 Ferrara, Italy

**Keywords:** *Neochloris oleoabundans*, Microalgae, Anti-inflammatory, Antioxidant, Cell morphology, Cultivation modes

## Abstract

Microalgae are considered promising sustainable sources of natural bioactive compounds to be used in biotechnological sectors. In recent years, attention is increasingly given to the search of microalgae-derived compounds with antioxidant and anti-inflammatory properties for nutraceutical or pharmacological issues. In this context, attention is usually focused on the composition and bioactivity of algae or their extracts, while less interest is driven to their biological features, for example, those related to morphology and cultivation conditions. In addition, specific studies on the antioxidant and anti-inflammatory properties of microalgae mainly concern *Chlorella* or *Spirulina*. The present work was focused on the characterization of the Chlorophyta *Neochloris oleoabundans* under two combinations of cultivation modes: autotrophy and glucose-induced mixotrophy, each followed by starvation. Biomass for morphological and biochemical characterization, as well as for extract preparation, was harvested at the end of each cultivation phase. Analyses indicated a different content of the most important classes of bioactive compounds with antioxidant/anti-inflammatory properties (lipids, exo-polysaccharides, pigments, total phenolics, and proteins). In particular, the most promising condition able to prompt the production of antioxidant algal biomass with anti-inflammatory properties was the mixotrophic one. Under mixotrophy, beside an elevated algal biomass production, a strong photosynthetic metabolism with high appression of thylakoid membranes and characteristics of high photo-protection from oxidative damage was observed and linked to the overproduction of exo-polysaccharides and lipids rather than pigments. Overall, mixotrophy appears a good choice to produce natural bioactive extracts, potentially well tolerated by human metabolism and environmentally sustainable.

**Supplementary Information:**

The online version contains supplementary material available at 10.1007/s00709-024-01958-7.

## Introduction

**Fig. 1:**
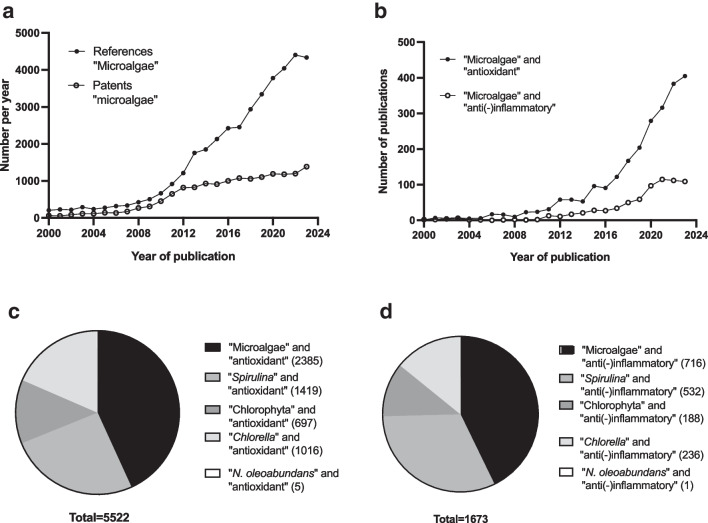
Number of documents on microalgae over 2000–2023 interval. **a** Number of patents (white circle) and references (black circle) per year between 2000 and 2023 using the only keyword “microalgae.” **b** Number of references per year between 2000 and 2023 using the keywords “microalgae” AND “anti(-)inflammatory” (white circle) or “antioxidant” (black circle). **c**, **d** Cake graphs showing total references from 2000 to 2023 using “microalgae” or “*Spirulina*” or “Chlorophyta” or “*Chlorella*” or “*Neochloris oleoabundans*” AND “antioxidant” (**c**) or “anti(-)inflammatory” (**d**) as keywords. Source: Scopus (03 January 2024)

Microalgae is a group of a wide range of photosynthetic microorganisms, both prokaryotic and eukaryotic ones; in nature, they are found in fresh, brackish, and marine waters, but also in extreme environments, like soils, rocks, springs, acidic lakes, or ice (Seckbach and Oren 2007; Pierre et al. 2019; do Carmo Cesário et al. 2022). To cope with such variable environments, microalgae have evolved an extensive range of morphological, physiological, and biochemical features, which make them promising natural and sustainable resources for biotechnological applications, like bioremediation, organic agriculture, and green energy production up to the health sector (Lauritano et al. 2016; dos Souza et al. 2017; Gaignard et al. 2019; Nethravathy et al. 2019; Pierre et al. 2019; Baldisserotto et al. 2020, 2023a; Fernandes and Cordeiro 2021; Mehariya et al. 2021). For the latter, microalgae are reported to be sustainable sources of natural bioactive compounds, such as pigments (mainly carotenoids), polyphenolics, polysaccharides, polyunsaturated fatty acids, vitamins, and small peptides (Tabarzad et al. 2020; Sadvakasova et al. 2023; Sirohi et al. 2021). Overall, interest in microalgae, in terms of biology and biotechnological applications, has grown rapidly in recent years as evidenced by both the number of scientific publications and the number of patents filed per year (Fernandes and Cordeiro 2021; Yang et al. 2023) (Fig. 1a).

With the aim of expanding the range of naturally derived bioactive products to replace synthetic ones in the nutraceutical or pharmacological fields, the attention is increasingly focused on the search for natural microalgae-derived compounds with antioxidant and anti-inflammatory properties (Lauritano et al. 2016; Assunçao et al. 2017; Choo et al. 2020; Conde et al. 2021; Coulombier et al. 2021; Goshtasbi et al. 2023) (Fig. 1). In particular, attention is mainly focused on the composition and bioactivity of algae or their extracts, while less attention is paid towards the importance of the biological features of the algae; for example, Almendinger et al. (2021) and Conde et al. (2021) characterized a good number of microalgae in terms of chemical composition and antioxidant properties, but with no attention to overall morphology and physiological responses of the algae or also their growth, for example, in relation with the cultivation environment. However, it is reported that cell morphology can give a fast indication of the abundance within microalgal cells of antioxidant/anti-inflammatory molecules, such as carotenoids (Solovchenko 2013; Tabarzad et al. 2020; Ávila-Román et al. 2021); in this sense, Schüler et al. (2020) found that *Tetraselmis* sp. accumulates lutein and β-carotene in the thylakoid membrane rather than in lipid droplets, unlike *Haematococcus pluvialis* or *Dunaliella salina*, which, instead, accumulate astaxanthin and β-carotene in lipid bodies (Davidi et al. 2014; Ota et al. 2018). This example supports the importance of combining biochemical and morpho-physiological features of algae strains to predict and explain bioactivity of extracts derived from microalgal biomass. On the other hand, literature is rich in examples on the impact of cultivation modes on the growth and the morphological, physiological, and biochemical response of microalgae, with relatively little attention on their bioactivity (Giovanardi et al. 2013; George et al. 2014; Rashidi et al. 2019; Rugnini et al. 2020; Al Jabri et al. 2021; Yun et al. 2021; Pandey et al. 2023; Wood et al. 2023). Particularly, the cultivation of microalgae can be manipulated by humans to favor the production of specific bioactive compounds by modifying the culture media or cultivation parameters; for example, nutrient starvation usually reduces growth, but promotes the accumulation of carbon skeletons in the form of lipids or starch, as observed for *Chlorella zoofingensis* or several other microalgae (Zhu et al. 2016; Shibzukhova et al. 2023). However, the relation between cultivation mode, antioxidant/anti-inflammatory properties, and morpho-physiology of algae remains less thorough (George et al. 2014; Pancha et al. 2014; Rugnini et al. 2020; Baldisserotto et al. 2021; Yun et al. 2021; Shibzukhova et al. 2023).

Among cultivation protocols, one strategy to promote algal growth and concomitant production of molecules of interest, such as lipids or cell wall polysaccharides, is the addition of organic carbon compounds into the culture media, thus inducing the mixotrophic metabolism. In such case, mixotrophic microalgae exploit light and both organic and inorganic carbon as energy sources for their growth (Villanova et al. 2017; Castillo et al. 2021; Yun et al. 2021; Abreu et al. 2022; Ma et al. 2023), thus improving biomass production yields and driving metabolism towards the enhanced production of molecules different to those normally produced under autotrophic conditions, lipids or also polysaccharides (Baldisserotto et al. 2016, 2021; Li et al. 2020; Yun et al. 2021). Another well-recognized way to modify microalgae morpho-physiology for biotechnological aims is to expose them to stressful conditions, such as starvation, cold/heat, or high light (Stirk et al. 2018; Chokshi et al. 2020; Rugnini et al. 2020; Shibzukhova et al. 2023). The sequential combination of different cultivation methods is often proposed and tested to emphasize the production of algal biomass enriched in different molecules of interest (Aziz et al. 2020; Ding et al. 2019). One example is the two-step cultivation, based on a first cultivation in a medium/condition promoting algal growth (for example, autotrophic standard conditions), followed by a second step in which the algae biomass is exposed to a stress condition (for example, starvation or high light stress) (Baldisserotto et al. 2014; Ding et al. 2019; Aziz et al. 2020). In all cases, attention is mainly focused on biomass production yields and the biochemical composition of algae, without an integrated investigation of algae morpho-physiology, cultivation aspects, and possible applications.

Since the present work deals with overall health care aspects linked to antioxidant and anti-inflammatory properties of algae extracts, it is important to underline some basic information about oxidative stress and inflammation. In this context, it is recognized that the oxidative stress can cause a damage to tissues, which can trigger an inflammation response and, in turn, directly prompt oxidative stress in a sort of a loop (Seyedsadjadi and Grant 2021). It is well established that unhealthy lifestyles can activate oxidative and inflammatory processes playing as primary drivers of the cell and tissue damage, and favoring the development, over time, of non-communicable diseases (NCD), such as Alzheimer’s, diabetes, cardiovascular disease (WHO 2017; Seyedsadjadi and Grant 2021). To improve lifestyle and help to prevent NCDs, one option is the introduction of antioxidant compounds in the diet, because the regular consumption of these products can contribute to the elimination of free radicals in the body (Ayoka et al. 2022). The possibility to substituting synthetic antioxidants with natural ones, such as those produced by microalgae, represents an added value, if one considers that the protracted intake of synthetically obtained antioxidants can raise safety issues (Kornienko et al. 2019; Velázquez-Sámano et al. 2019; Gauthier et al. 2020). As briefly hinted at above, the production of antioxidant and anti-inflammatory molecules by microalgae is attracting increasing interest in the research community (Fig. 1b). However, the subject area remains still open, especially considering the very wide variety of microalgae and the great diversity of their metabolic responses depending on the environment in which they grow naturally or are artificially cultivated by humans (Metting 1996; Izadpanah et al. 2018; Gaignard et al. 2019; Sansone and Brunet 2019; Fernandes and Cordeiro 2021; www.algaebase.org website).

Among microalgae, *Neochloris oleoabundans* (Chlorophyta) has been extensively studied since the 1980s for its ability to produce lipids for biodiesel production (Tornabene et al. 1983; Baldisserotto et al. 2016; Abu-Hajar et al. 2017; Khan et al. 2022). Thanks to these studies, also the feasibility of cultivating the alga in large volumes, for example in photobioreactors, has been quite widely explored, thus suggesting a possible exploitation of *N. oleoabundans* for actual applications (Pruvost et al. 2009; Sabia et al. 2015; Norsker et al. 2021). Furthermore, more recently the alga has been studied also for human health and well-being purposes, concerning in detail bioactive carotenoids with anti-proliferative activity (mainly violaxanthin; Castro-Puyana et al. 2017), content in extracellular polysaccharides with immunomodulatory properties (EPS1 isolate, formed by 0.59% peptides and glucose, mannose and galactose as main monosaccharides; Li et al. 2020), use in the formulation of cosmetics (Morocho-Jácome et al. 2022), and anti-viral properties (Baldisserotto et al. 2023a). Other few studies are available on the antioxidant properties of the alga, but merely in the context of microalgae screening or for technical optimization of extraction procedures (Goiris et al. 2012; Castro-Puyana et al. 2013; Banskota et al. 2019). Overall, it emerges that *N. oleoabundans*, while being extensively investigated for the bioenergetic application, is still poorly studied with regard to its antioxidant and anti-inflammatory properties if compared to other microalgae, like *Chlorella* or *Spirulina*, or to Chlorophyta in general (Fig. 1c, d). Furthermore, no information is available on the possible relation between cell morphology and antioxidant and anti-inflammatory properties of the alga under different cultivation modes.

In particular, for this work, we chose to approach an experimental study on *N. oleoabundans* cultivated under two combinations of cultivation modes, in which the first step of cultivation in autotrophic or mixotrophic conditions was followed by a second step of cultivation in nutrient starvation. Algae were evaluated in terms of growth capability and physiological responses of the photosynthetic apparatus, with emphasis on their morphology and biochemical composition. Algae were then employed to produce aqueous extracts, which were tested for their antioxidant and anti-inflammatory properties using murine microglial cells. With this work, we expect to better define morphological peculiarities, basic biochemical composition, and the potential of *N. oleoabundans* as source of antioxidant/anti-inflammatory extracts. Summarizing, in a biotechnological application perspective, to identify the best cultivation mode for obtaining algal biomass with effective bioactivity, our main intention was aimed at understanding how different cultivation condition protocols may affect the overall status of *N. oleoabundans*, and then, how much the biological response of the alga impacts the antioxidant/anti-inflammatory activity of its extracts.

## Materials and methods

### Algal material and experimental design

The green microalga *Neochloris oleoabundans* (syn. *Ettlia oleoabundans*) strain UTEX-1185 from the Culture Collection of Algae of the University of Texas at Austin (USA; www.utex.org) was used to produce 4 whole microalgal extracts for antioxidant and anti-inflammatory tests. Extracts were obtained at the end of the cultivation period from autotrophic and mixotrophic cultures, also after their starvation in tap water, in 2-stage cultivation systems. Harvesting time of algae for extracts preparation was different based on the cultivation mode. Both the types of algal culture and the extracts derived from the corresponding cultivation are defined as follows: A, SA, M, and SM, respectively for autotrophic, starved autotrophic, mixotrophic, and starved mixotrophic cultures or extracts.

For autotrophic cultures (A), aliquots of dense, starter cultures of *N. oleoabundans* cultivated in BG11 synthetic mineral medium (pH 7.1; for recipe, see Table S1; utex.org/products/bg-11-medium) were inoculated in fresh medium to give a final cell density of about 0.5 × 10^6^ cells mL^−1^ in 500-mL Erlenmeyer flasks (300 mL of culture volume). Algae were grown for 28 days in a growth chamber (24 ± 1 °C, 80 μmol_photons_ m^−2^ s^−1^ of photosynthetically active radiation (PAR—400 to 700 nm wavelength -, measured with a quantum photoradiometer mod. HD9021, Delta Ohm, Caselle di Selvazzano (Padova), Italy) with a 16:8-h light-darkness photoperiod), with continuous shaking using an orbital shaker (VDRL, mod 711D, Asal srl, Cernusco sul Naviglio (Milano), Italy) set at 110 rpm without CO_2_ addition, and then, they were employed to produce a whole aqueous extract (extract A; for the method, see Paragraph “Preparation of whole extracts for antioxidant and anti-inflammatory tests”). For starved autotrophic cultures (SA), a part of cultures A was harvested by centrifugation (8000 g, 10 min) at the end of the cultivation period (28 days) and then was resuspended in an equal volume of sterilized tap water (tap water composition is available at the website of the local agency for water, energy, and environment management (HERA—Holding Energia Risorse Ambiente), and is reported in Table S2). These cultures were maintained in the same chamber described above for additional 28 days; thereafter, they were used for extracts preparation (extract SA). For mixotrophic cultures (M), aliquots of dense, starter cultures of *N. oleoabundans* maintained in BG11 medium were inoculated in fresh medium added with 2.5 g L^−1^ of sterilized glucose (Merck; stock solution: 50 gL^−1^ in BG11 medium) to give a final cell density of about 0.5 × 10^6^ cells mL^−1^ as for cultures A; cultures were kept for 12 days in the growth chamber at the same conditions described above, and then, they were harvested for the preparation of extract M. Finally, a part of these mixotrophic cultures were harvested by centrifugation (8000 g, 10 min) at the end of the cultivation time (12 days), and the algal pellet was then resuspended in an equal volume of sterilized tap water in order to obtain starved mixotrophic cultures (SM); these cultures were kept in the growth chamber for further 20 days and subsequently were used for the preparation of extracts SM. For starved cultures, prolonged cultivation in nutrient limitation conditions was chosen due to previous studies on *N. oleoabundans*, which showed evident morphological modifications, including lipid accumulation, after 21–28 days of nutrient starvation (Giovanardi et al. 2013; Baldisserotto et al. 2014); furthermore, it is reported that bioactive metabolites are produced over time under starvation conditions (Solovchenko et al. 2008).

The choice of the organic carbon source and harvesting times for cultures were based on previous findings. In detail, it is reported that *N. oleoabundans* prefers glucose to other organic carbon sources (sucrose, fructose, acetate, maltose, galactose) (Giovanardi et al. 2013; Li et al. 2020). It is indeed well-known that the organic carbon metabolism is species-dependent, so that the use of different organic carbon substrates leads to different growth results (Pang et al. 2019; Baldisserotto et al. 2021; Gulk et al. 2023). As regards harvesting algal biomass at the stationary phase of growth for autotrophic and mixotrophic cultures, but also after prolonged starvation times for the second phase of cultivation, it was linked to literature works which assessed that proper prolonged cultivation combines the goal of obtaining the highest biomass with the highest accumulation of metabolites with bioactive properties (Baldisserotto et al. 2014, 2023a; Giovanardi et al. 2013; Ribeiro et al. 2022). Furthermore, it is reported that bioactive metabolites are produced over time under starvation conditions (Solovchenko et al. 2008).

Algae cultures A and M were set up in triplicate in two independent experiments, so that 3 replicates of them were used to obtain cultures SA and SM after starvation in tap water (i.e., the 2nd cultivation stage of A and M cultures). All algal samples were handled in sterile conditions. During cultivations, cell density and photosystem II (PSII) maximum quantum yield of algae were routinely assessed to monitor their growth and overall physiological behavior. At the end of each cultivation period (i.e., when algal biomass was harvested for extracts preparation), dry biomass yield, photosynthetic pigments, total protein, and total phenolic contents of the algae were evaluated, as well as observations of their morphology and ultrastructure were done through light and transmission electron microscopy.

### Cell density and dry biomass yield evaluation

The cell densities of all cultures (million cells per mL; 10^6^ cells mL^−1^) were evaluated using a Thoma’s counting chamber (HBG, Giessen, Germany). Cell densities were checked at different experimental times depending on the type of cultivation mode and plotted on a logarithmic scale to obtain the respective growth kinetics. On the basis of cell densities, growth rates (μ; d^−1^) were calculated (Baldisserotto et al. 2023b). Furthermore, at the end of the cultivation periods, the dry biomass yield (i.e., grams of algal dry weight, DW, per liter; g_DW_ L^−1^) was evaluated for all algal samples as reported in Baldisserotto et al. (2016, 2023b). Briefly, aliquots of algal cultures were filtered using pre-weighed and pre-dried glass fiber filters (Whatman GF/C; 1.2-μm pore size); then, filters were rinsed with distilled water, dried for 72 h at 60 °C in an oven and weighted with an Ohaus analytical balance until constant weight was reached.

### PSII maximum quantum yield evaluation

For the evaluation of the maximum yield of PSII (F_*V*_/F_*M*_ ratio), a junior pulse amplitude modulation (PAM) fluorimeter was employed (Walz, Germany). For the measurements, aliquots of cultures were harvested by centrifugation at 10,000 g for 5 min. The pellet was resuspended in a small aliquot of the supernatant, gently deposited, drop by drop, onto a wet chromatographic paper (Whatman; 1 × 4 cm), and then dark-adapted for 15 min before fluorescence measurements (Ferroni et al. 2018). Basal and maximum fluorescence (F_*0*_ and F_*M*_, respectively) values were measured flashing the samples with a saturating light pulse (0.6 s), and then used for the calculation of the maximum quantum yield of PSII as F_*V*_/F_*M*_ ratio, where variable fluorescence F_*V*_ is F_*M*_-F_*0*_ (Kalaji et al. 2014).

### Photosynthetic pigment extraction and quantification

At the end of cultivation periods, just before whole aqueous extracts preparation, aliquots of cell samples were harvested by centrifugation (8000 g, 10 min), and the extraction of photosynthetic pigments was performed with absolute methanol according to Baldisserotto et al. (2023a). The extracts were manipulated under dim green light to avoid pigment photo-degradation. Optical density of extracts was measured with a Pharmacia Ultrospec 2000 UV–Vis spectrophotometer (1-nm bandwidth; Amersham Biosciences, Piscataway, NJ, USA) at 665 nm (Chlorophyll *a*, Chl*a*), 653 nm (Chlorophyll *b*, Chl*b*), 470 nm (Carotenoids, Car), and 750 nm (background noise). Quantification of pigments concentration was performed as reported in Wellburn (1994) and expressed as a percentage of total dry weight (%DW). The molar ratio of Chl*a* over Chl*b* and of total Chls over carotenoids was also evaluated based on pigments content per cell.

### Total protein extraction and quantification

The total protein content of algae in the 4 different cultures used for whole extracts preparation was evaluated according to Baldisserotto et al. (2023a). In detail, aliquots of algal samples were centrifuged for 10 min at 500 g and the pellets were resuspended with 2 mL of washing buffer (2 mM Na_2_EDTA in PBS buffer 1 × ; for 1L of 10 × PBS buffer stock solution: 80 g NaCl, 2 g KCl, 14.4 g Na_2_HPO_4_ × 2H_2_O, 2.4 g KH_2_PO_4_ dissolved in distilled water), transferred into Eppendorf tubes, and then centrifuged (2000 g, 10 min) (washing step). Subsequently, pellets were resuspended in extraction buffer (0.1 M NaOH, 1% sodium dodecyl sulfate, 0.5% β-mercaptoethanol dissolved in distilled water), frozen in liquid N_2_ for 2 min and then heated in a thermostatic bath set at 80 °C for other 2 min (3 times per cold-hot cycle). Thereafter, samples were rapidly frozen in liquid N_2_ and kept at – 20 °C overnight. On the following day, the samples were added with glass beads (0.40–0.60 μm diameter; Sartorius, Göttingen, Germany) and vigorously vortexed for 10 min (mixing cycles of 30 s followed by cooling on ice for 30 s). Then, the samples were centrifugated (1500 g, 10 min) and the supernatants (1st proteins extract) were harvested. Subsequently, the pellets remaining inside the tubes were re-extracted by resuspending with 0.5 mL of the same extraction buffer described above; tubes were then vortexed for 2 min nonstop and subsequently heated at 60 °C for 15 min. After centrifugation (1500 g, 10 min), supernatants were added to the 1st proteins extract, rapidly frozen in liquid N_2_ and kept at − 20 °C until quantification. The quantitative estimation of proteins, expressed as percentage of total dry weight (%DW), was determined with a modified Lowry’s method (Lowry et al. 1951), using bovine serum albumin (BSA) as the standard (Sigma Chemicals, USA).

### Total phenolic extraction and quantification

For total phenolics extraction, aliquots of algal biomass containing about 120 × 10^6^ cells were harvested by centrifugation (600 g, 10 min) and stored at – 20 °C until extraction. Extracts preparation was performed according to Haoujar et al. (2019) with minor modifications. In detail, algal biomass was mixed with 100% methanol and then exposed to mechanical disruption (15 min of nonstop vortexing, followed by 30 min incubation in an ultrasound bath, and further 10 min-long vortexing). After extraction, samples were centrifugated (1500 g, 10 min), and the supernatants were harvested for the quantification with the Folin-Ciocalteu reaction as described in Baldisserotto et al. (2023a). The total phenolics concentration in the extracts was calculated on the basis of a calibration curve prepared using coumaric acid (Sigma Chemicals, USA) as the standard. Results are expressed as mg_Eq_ of coumaric acid over g of algal dry weight (mg_Eq Cum.Ac._ g_DW_^−1^).

### Transmission electron microscopy (TEM)

For TEM observations, aliquots of algal samples were harvested by centrifugation (600 g, 10 min) and processed as reported in Baldisserotto et al. (2020, 2023a). For observations, a transmission electron microscope Zeiss EM910 was employed (Electron Microscopy Center, University of Ferrara, Ferrara, Italy).

### Light and epifluorescence microscopy

Morphology of algae was observed with a Zeiss, model Axiophot photomicroscope equipped with an epifluorescence apparatus. The light source for chlorophyll (Chl) fluorescence observation was an HBO 100-W pressure mercury vapor lamp (filter set, BP436/10, LP470).

The intracellular presence of lipids was evaluated by staining cells with the fluorochrome Nile Red (9-diethylamino-5Hbenzo[α]phenoxazine-5-one, 0.5 mg dissolved in 100 mL acetone) (Sigma-Aldrich, Gallarate, Milan, Italy), as described in Giovanardi et al. (2013). For stained samples, the excitation wavelength of 485 nm (filter set, BP485, LP520) was used to highlight the presence of lipid droplets, emitting a yellowish-gold fluorescence (Giovanardi et al. 2013). Differently, the production and release of extracellular polymeric substances (EPS), mainly formed by exo-mucopolysaccharides, from algae were observed using Alcian Blue 8GX (Serva) dye, prepared at 1% in 3% acetic acid, pH 2.5 (Mowry and Scott 1967; Fagan et al. 2020). In detail, 500 μL of algae samples were harvested by centrifugation (10,000 g, 5 min), and then, 20 μL of dye solution was added to the pellet; the reaction was carried out for 30 min at room temperature (RT). After staining, cells were washed with distilled water to remove excess dye and then observed with the aforementioned Zeiss microscope using conventional light. The blue coloration conferred by the dye indicates the presence of acidic exo-mucopolysaccharides produced at the cell wall level in the algal cells and/or released by the cells (Discart et al. 2015; Vergnes et al. 2018).

Photographs were taken with a VisiCam Pro 20C digital camera (20 megapixels) mounted on an ocular adaptor.

### Preparation of whole extracts for antioxidant and anti-inflammatory tests

Algal biomass was harvested by centrifugation (600 g, 10 min) from all samples at the end of the cultivation periods and then it was washed with distilled sterile water. Cells were mechanically broken in a cold pre-sterilized ceramic mortar in the presence of sand quartz (40–100 mesh; Fisher Chemicals) and liquid N_2_, and then resuspended to a final concentration of 25g_AlgDW_ L^−1^ in the sterile medium used for cultivation of microglia cells, to avoid interferences during tests on cell lines (see Paragraph below “Cell Cultures”). The resulting whole extracts were centrifuged to remove cell debris, filter sterilized (PVDF syringe filters, 0.2-μm pore size) and stocked in small aliquots at 4 °C for subsequent tests on antioxidant and anti-inflammatory activities.

### Cell cultures

N9 murine microglial cells were grown in Iscove’s modified Dulbecco’s medium (IMDM) with 5% heat-inactivated Australian FBS (fetal bovine serum), 1% penicillin (100 U mL^−1^), and streptomycin (100 μg mL^−1^). Cells were kept in a humidified environment, with 5% of CO_2_ and 37 °C of temperature, and were diluted three times a week to maintain the optimal confluence (80%).

### Cellular treatments

N9 cells were stimulated with 1 μg mL^−1^ of lipopolysaccharide (LPS) (from *Escherichia coli*, serotype 055:B5, soluble in cell culture medium) for 24 h in order to trigger the pro-inflammatory response. Algal extracts, tested at different dilutions, were added 30 min before LPS to cells cultured in serum free medium.

### DPPH test

The antioxidant capacity of 1:5 dilutions of algal extracts was tested with 2,2-diphenyl-1-picrylhydrazyl (DPPH) assay according to Tedeschi et al. (2023). In detail, each tested extract and the ascorbic acid were added, in duplicate, in a black 96 well-plate containing 0.1 mM DPPH or methanol for the blank. The 96 well-plate was mixed for 30 min in an orbital shaker in the dark at room temperature. Then, the absorbance was measured with an Ensight multimodal plate reader (Perkin Elmer, Milan) at 517 nm. The antioxidant ability was calculated as percentage of inhibition versus ascorbic acid (50 μM), used as positive control.

### MTS assay

The MTS assay was performed to determine cells vitality according to the manufacturer’s protocol from the CellTiter 96 Aqueous One Solution cell proliferation assay (Promega, Milan, Italy). Cells were plated in 96-multiwell plates (30,000 cells/well), allowed to attach overnight; then, 100 μL of complete medium was added to each well in the absence and in the presence of the algal extracts for 24 h. At the end of the incubation period, MTS solution was added to each well. The optical density of each well was read with the Ensight multimodal plate reader cited above at 570 nm.

### Nitric oxide assay

The anti-inflammatory potential of algal extracts was tested in N9 microglial cells with the Nitrate/Nitrite Colorimetric Assay Kit (Merighi et al. 2021). Nitrate/Nitrite Colorimetric Assay Kit was purchased by Vinci-Biochem (Florence, Italy). In detail, 150,000 cells were seeded in a 24 wells plate and incubated for 24 h, 80 μL of the supernatants of each well were transferred to a 96 well plate with 10 μL of the nitrate reductase and 10 μL of its cofactor. After 2 h of incubation, the two Griess reagents were added, converting the total nitrite to a purple azoic compound. The absorbance measurement was performed with the abovementioned Ensight multimodal plate reader set at 550 nm. The standard curve was performed with nitrate, allowing the determination of the nitrate + nitrite concentration, which is proportional to the red absorbance.

### Statistical data treatment

Data were processed with Graphpad Prism 9 (Graph Pad Software, San Diego CA, USA) and are reported as means ± standard deviations (s.d.) for *n* number of samples. The statistical significance of differences was determined by one-way ANOVA followed by a multiple comparison test (Tukey’s test or Dunnett’s test). A significance level of 95% (*p* < 0.05) was accepted. For results on growth rates, Student’s *t* test was performed (significance level, 0.05).

## Results

### Growth kinetics and PSII maximum quantum yield of *N. oleoabundans* under two-stages cultivation modes

**Fig. 2:**
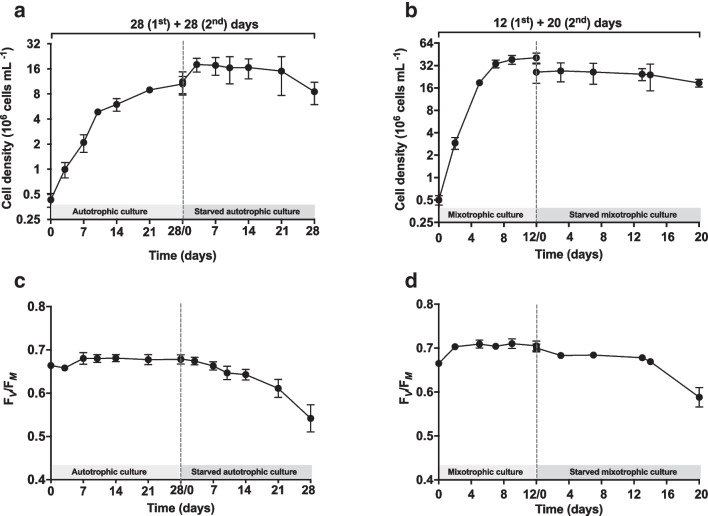
Growth kinetics (10^6^ cells mL^−1^; **a**, **b**) and trend of the maximum quantum yield of PSII (F_*V*_/F_*M*_; **c**, **d**) of *N. oleoabundans* cultures. **a**, **c** Autotrophic cultures grown for 28 days (1st stage), followed by 28 day-long starvation (2nd stage). **b**, **d** Mixotrophic cultures grown for 12 days (1st stage), followed by 20 day-long starvation (2nd stage). Inside all graphs, the dotted line indicates the switch of cultures from 1st stage autotrophic (**a**, **c**) or mixotrophic (**b**, **d**) conditions to 2nd stage cultivation under starvation in tap water. Data refer to mean with standard deviations (*n* ≥ 3)

For all samples, growth kinetics and maximum yield of PSII were monitored to assess the overall status of algae during cultivations (Fig. 2).

Autotrophic samples A, which were set up at a cell density of about 0.5 × 10^6^ cells mL^−1^ at the inoculation time, were characterized by a first, evident 10 day-long period of the exponential phase of growth, followed by a second, slower growth period between 10 and 28 days of cultivation. Accordingly, these two phases (time intervals, 0–10 and 10–28 days) gave growth rates of 0.35 and 0.06 day^−1^, respectively (*p* < 0.001) (Fig. 2a). As expected, samples SA started from a higher cell density (about 11 × 10^6^ cells mL^−1^), because they derived from cultures A at the 28th day of cultivation, then harvested and resuspended in tap water. In line with expectations, SA algae showed a limited growth only between 0 and 4 days of cultivation in tap water (*μ* = 0.18 day^−1^); subsequently, the cultures interrupted their growth (4–21 days) and their cell density even decreased during the last week of cultivation (21–28 days) (Fig. 2a).

Differently, mixotrophic samples M showed a very fast growth, which lasted only 12 days: an evident exponential phase of growth lasted from 0 to 7 days of cultivation (μ = 0.81 day^−1^), and then, the stationary phase was reached with a cell density of about 38–40 × 10^6^ cells mL^−1^ (Fig. 2b). After changing the cultivation condition, i.e., when stationary mixotrophic cells were harvested and resuspended in tap water for the 2nd stage cultivation, the cell density of SM cultures started with a value around 26 × 10^6^ cells mL^−1^ due to partial loss of algae biomass during the harvesting activities. In SM cultures, the cell density remained unchanged at substantially stable values of around 24–26 × 10^6^ cells mL^−1^ up to 14 d, then it decreased to values around 18 × 10^6^ cells mL^−1^ at the end of the cultivation period (20 days), resulting in an overall negative growth rate (μ =  − 0.03 day^−1^) (Fig. 2b).

Analogous to growth kinetics, under A conditions, F_*V*_/F_*M*_ values of cultures maintained stable values around 0.66–0.68, while an evident, gradual decrease occurred in SA samples (from 0.680 to 0.542, respectively at 0 and 28 days of starvation); the decrease was particularly pronounced from day 21 to 28 of cultivation in tap water (Fig. 2c). Differently, M samples started with F_*V*_/F_*M*_ values of about 0.66 at time 0 and then they increased to values around 0.7, which was maintained up to up the 12th cultivation day (end of cultivation; Fig. 2d). The starvation period was characterized by algae with quite stable F_*V*_/F_*M*_ values of about 0.68 up to 14 days; then, the parameter apparently decreased down to about 0.59 at the 20th day of cultivation (Fig. 2d).

### Characteristics of *N*. *oleoabundans* cultures used for extracts preparation

**Fig. 3:**
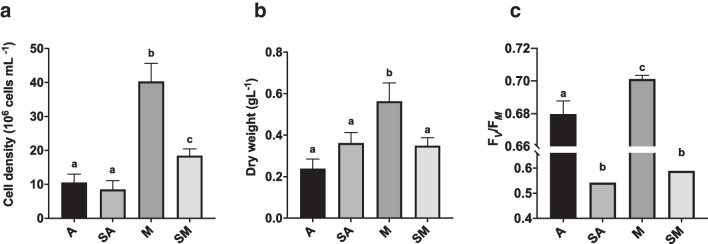
Evaluation of cell density (10^6^ cells mL^−1^; **a**), dry biomass yield (g_DW_ L^−1^; **b**) and PSII maximum quantum yield (F_*V*_/F_*M*_ ratio; **c**) of *N. oleoabundans* cultures used for extracts preparation. A, autotrophic cultures (harvesting time: 28 days); SA, starved autotrophic cultures (harvesting time: 28 days); M, mixotrophic cultures (harvesting time: 12 days); SM, starved mixotrophic cultures (harvesting time: 20 days). Data refer to means with standard deviations (*n* ≥ 3). Different letters refer to statistically different samples after the one-way ANOVA test followed by Tukey’s post hoc analysis (*p* < 0.05)

**Fig. 4:**
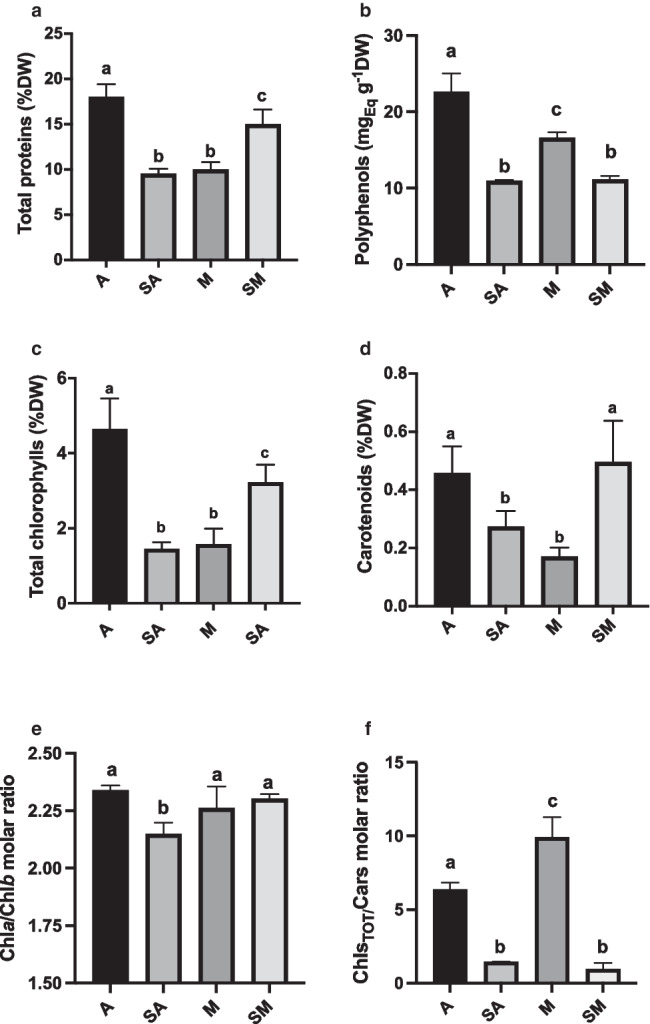
Composition and photosynthetic pigments molar ratios of *N. oleoabundans* algae samples employed for extract preparation. **a** Total proteins, **b** phenolics, **c** chlorophylls, **d** carotenoids contents, **e** Chl*a* over Chl*b* molar ratio, and **f** total chlorophylls over carotenoids molar ratio. In **a**, **c**, **d**, concentrations are expressed as %DW, while in **b** as mg_Eq Coum. Ac__._ g_DW_^−1^. A, autotrophic cultures (harvesting time: 28 days); SA, starved autotrophic cultures (harvesting time: 28 days); M, mixotrophic cultures (harvesting time: 12 days); SM, starved mixotrophic cultures (harvesting time: 20 days). Data refer to means with standard deviations (*n* ≥ 3). Different letters refer to statistically different samples after the one-way ANOVA test followed by Tukey’s post hoc analysis (*p* < 0.05)

**Fig. 5:**
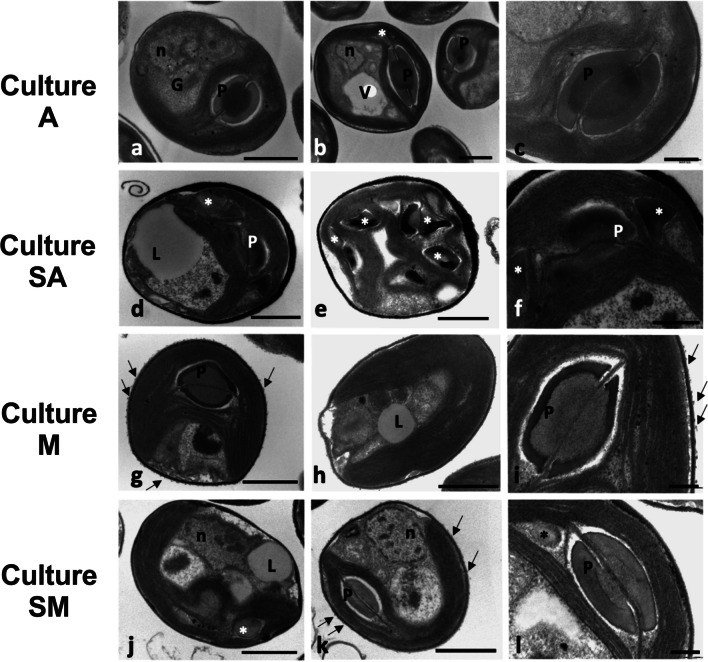
Transmission electron microscopy images of *N. oleoabundans* algae cells belonging to A (**a**–**c**), SA (**d**–**f**), M (**g**–**i**), and SM (**j**–**l**) cultures used for extracts preparation, where A refer to autotrophic cultures at time 28 days, SA to starved autotrophic culture harvested at time 28 days, M to mixotrophic cultures at 12 days of cultivation and SM to starved mixotrophic cultures harvested at time 20 days. P, pyrenoid; n, nucleus; L, lipid globule; G, Golgi apparatus; V, vacuolation; *, stromatic starch; arrows, flaky material extruded by the cell wall. Bars: **a**–**h**, **j**, k = 1 μm; **i**, **l** = 0.2 μm

**Fig. 6:**
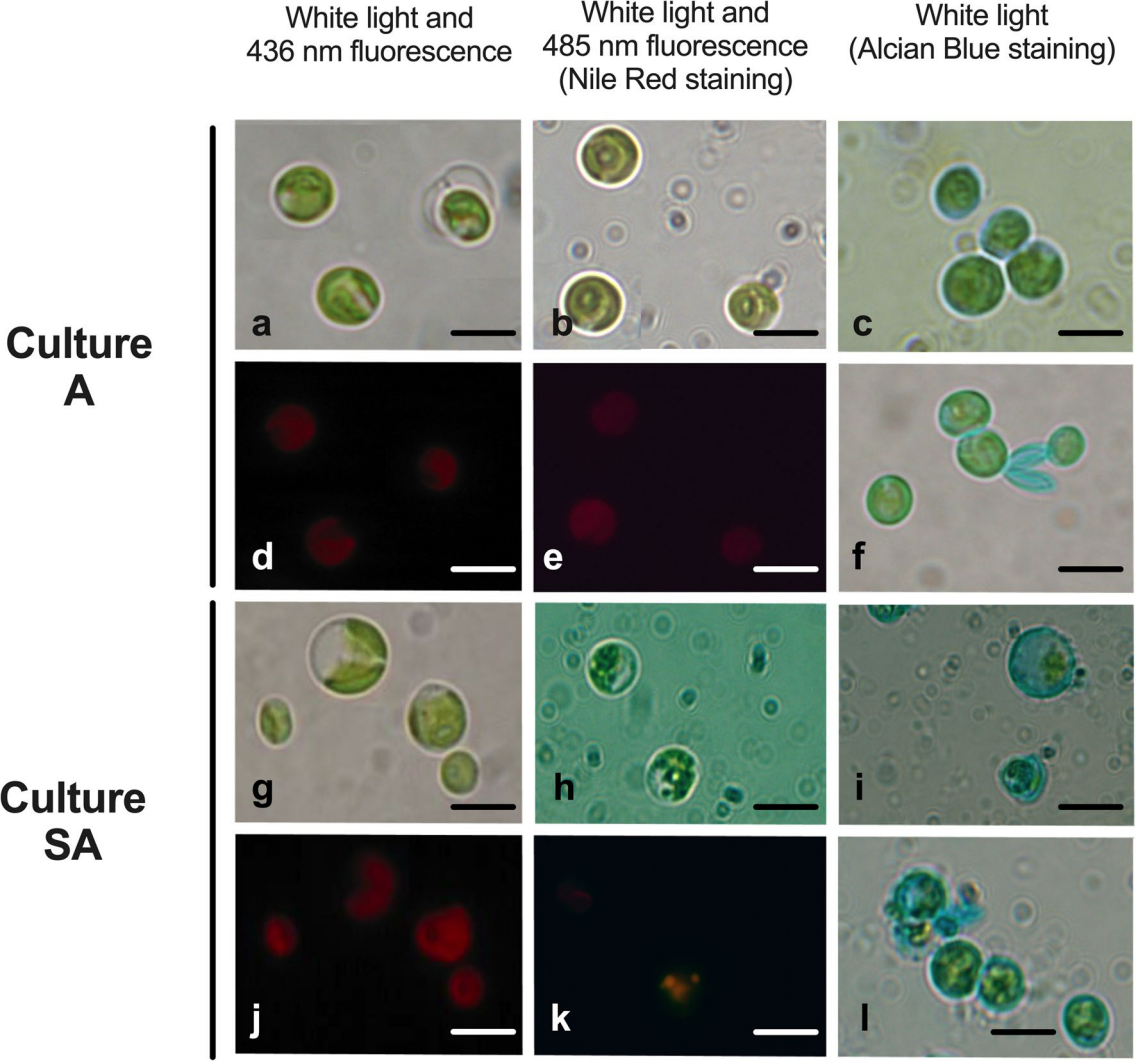
Light microscopy images of *N. oleoabundans* belonging to autotrophic A (**a**–**f**) and starved autotrophic SA (**g**–**l**) cultures. For both A and SA, samples refer to algae harvested at time 28 days of cultivation. The left column shows non-stained cells, while the central and right column the cells stained with Nile Red or Alcian Blue, respectively. In the left column, conventional light microscopy images are shown in (**a**, **g**) and chlorophyll autofluorescence under excitation at 436 nm (**d**, **j**). In the central column, **b**, **h** refer to images taken under conventional light, **e**, **k** under UV light at 485 nm of excitation. In the left column, all pictures (**c**, **f**, **i**, **l**) refer to images taken under conventional light. Arrows, pyrenoid. Bars: 3 μm

**Fig. 7:**
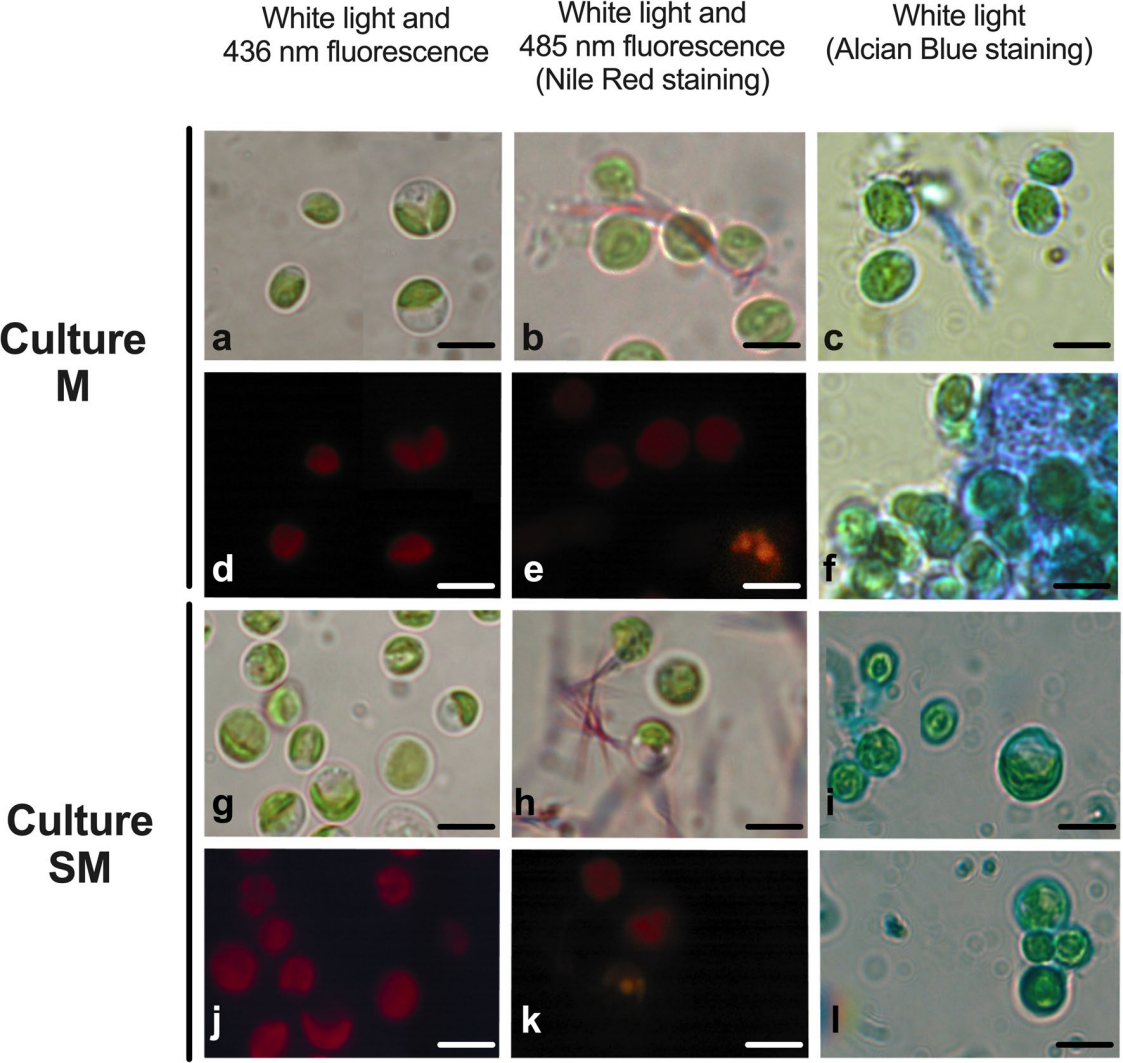
Light microscopy images of *N. oleoabundans* belonging to mixotrophic M (**a**–**f**) and starved mixotrophic SM (**g**–**l**) cultures. M and SM samples refer to algae harvested at time 12 and 20 days of cultivation, respectively. The left column shows non-stained cells, while the central and right column the cells stained with Nile Red or Alcian Blue, respectively. In left column, conventional light microscopy images are shown in (**a**, **g**) and chlorophyll autofluorescence under excitation at 436 nm in (**d**, **j**). In the central column, **b**, **h** refer to images taken under conventional light, **e**, **k** under UV light at 485 nm of excitation. In the left column, all pictures (**c**, **f**, **i**, **l**) refer to images taken under conventional light. Bars: 3 μm

Before extraction, algae samples from all cultures were characterized and compared as regards cell density, dry biomass, F_*V*_/F_*M*_ values, and their content in total proteins, photosynthetic pigments, and phenolics (Figs. 3 and 4). Parallelly, cell morphology and ultrastructure were evaluated (Figs. 5, 6, and 7). In detail, light microscopy observations, also performed after specific staining for lipids and EPS, were meant to both give an overall picture of cells and to highlight the presence of bioactive molecules, potentially responsible for antioxidant and anti-inflammatory activity. For all examinations, results refer to algae samples harvested at the end of each specific cultivation period: 28, 28, 12, and 20 days, respectively for A, SA, M, and SM samples.

#### Productivity, PSII photosynthetic performance, and biochemical characterization

At the end of cultivation, autotrophic and starved autotrophic cultures, respectively A and SA, were characterized by a similar cell density (about 10 and 8.5 × 10^6^ cells mL^−1^, respectively; *p* > 0.05) (Fig. 3a). In comparison, cell density of mixotrophic (M, 40.7 × 10^6^ cells mL^−1^) and starved mixotrophic (SM, 18.6 × 10^6^ cells mL^−1^) cultures was significantly higher than that obtained from both A and SA samples (ANOVA, *p* < 0.001; *F*(3,12) = 75.22) (Fig. 3a). In detail, M cultures had the highest cell density, while SM cultures, though showing lower values than those of M samples, were still about twice as concentrated as both A and SA cultures, thus resulting in the following sequence of cell densities: M > SM > A = SA (Fig. 3a). Interestingly, the dry biomass yield did not perfectly reflect the culture cell density, because SA samples had a higher, even if not significant, yield than A samples, and similar values to that of SM cultures. The highest dry biomass was obtained from M cultures. In detail, values were 0.56 g L^−1^ for M, and 0.35, 0.36, and 0.24 g L^−1^ for SM, SA, and A, respectively) (ANOVA, *p* < 0.001; *F*(3,12) = 20.90) (Fig. 3b). As expected, the quantum yield of PSII, which reflects the performance of PSII in the photosynthetic membranes and consequently the overall physiological status of algal cells, was higher in A and M samples than in starved SA and SM cultures, which were characterized by F_*V*_/F_*M*_ values below 0.6 (Fig. 3c) (ANOVA, *p* < 0.001; *F*(3,14) = 51.71). The highest value was recorded for M samples (about 0.70).

The chemical composition of algal biomass was evaluated as content of total proteins, total phenolics, and photosynthetic pigments (total chlorophylls and carotenoids) (Fig. 4). The highest concentration of total proteins, chlorophylls, and phenolics was obtained by samples A, which reached 18 and 4.6% on a dry weight basis, respectively for proteins and chlorophylls, and 22.6 mg_Eq Cum. Ac._ g_DW_^−1^ for phenolics, i.e., about 2.3% of the total dry biomass (Fig. 4a–c). Differently, for carotenoids, the highest concentration was recorded for both A and SM samples, with a percentage on dry weight of 0.45–0.49% (*p* > 0.05) (Fig. 3d). In detail, as compared with algae A, the total protein content of SA and M samples was approximatively halved, while for SM samples the difference was only around − 17% (ANOVA, *p* < 0.001; *F* (3,12) = 47.24) (Fig. 4a). A substantially similar trend occurred for total chlorophyll content, even though with larger differences (SA and M, 3 × lower values than A, while SM, − 30%; ANOVA, *p* < 0.001; *F* (3,13) = 41.32) (Fig. 4c). Differently, the carotenoid content was similar in A and SM samples, while it was lower in SA and M samples (differences ranged between − 40 and − 60%; ANOVA, *p* < 0.001; *F* (3,13) = 14.13) (Fig. 4d). Finally, as regards the total phenolics content, M samples had values 26% lower than those in sample A, while the lowest values were found in SA and SM samples, with halved values (around 11 mg_Eq Cum. Ac__._ g_DW_^−1^ in both cases) compared to A samples (ANOVA, *p* < 0.001; *F* (3,14) = 95.65) (Fig. 4b).

Interestingly, for photosynthetic pigments, the Chl*a*/Chl*b* molar ratio was not significantly different among samples A, M, and SM (ANOVA, *p* < 0.001; F (3,11) = 11.45), while the Chl_TOT_/Cars values were very different (A, 6.35; SA, 1.46; M, 9.90; SM, 1.06; ANOVA, *p* < 0.001; *F*(3,13) = 145.3) (Fig. 4e, f).

#### Morphological characterization

Observation of all algal samples with light and transmission electron microscopy (TEM) showed that the microalgae did not differ so much in shape and size, irrespective of the cultivation protocol employed (Figs. 5, 6, and 7). The cells were nearly spherical or slightly flattened: the spherical cells had a diameter of about 2.5–3 μm, and the flattened ones were characterized by the major and minor axes of about 2.5–3 μm and 2.2–2.7 μm, respectively (Figs. 5, 6, and 7).

#### Ultrastructural characterization

Focusing attention on the ultrastructure of control autotrophic *N. oleoabundans* cells A, typical organelles, like the chloroplast, the nucleus, and the Golgi apparatus, were visible in the ultrathin sections (Fig. 5a, b). Inside the large cup-shaped chloroplast, the characteristic pyrenoid was always evident, with its proteinaceous inner matrix crossed by one-two elongated thylakoid membranes and surrounded by a starch shell (Fig. 5a–c). Instead, the stromatic starch was scarce (Fig. 5b). The thylakoid membranes were typically grouped in elongated bundles for the entire length of the chloroplast (Fig. 5a, b). Some cells were vacuolated (Fig. 5b), while the cell wall uniformly surrounded all algae (Fig. 5a–c). Ultrastructure of *N. oleoabundans* from the other cultivations showed some differences, mainly due to the abundance of stromatic starch, the presence of lipid globules, and/or cell wall modifications (Fig. 5d–l). In detail, SA algae showed the most enlarged and irregular cell walls; in some cells, the cytoplasm contained pale lipid droplets and a chloroplast rich in stromatic starch, but seemingly no pyrenoid or alternatively an altered one, which was still crossed by thylakoids but surrounded by distorted matrix and starch; furthermore, waved, elongated thylakoids characterized the plastid (Fig. 5d–f). Mixotrophic M algae were characterized by a large cup-shaped chloroplast, with a typical pyrenoid and thylakoid membrane system, similarly to those found in autotrophic A samples (Fig. 5g–i). Different to algae A, some M cells contained pale globules, due to lipid accumulations, and their cell wall was covered by extruded electron-dense flaky material (Fig. 5g–i). In some cases, vesiculations were visible next to the plasma membrane (Fig. 4g). Finally, in algae belonging to SM samples, the large chloroplast contained elongated and appressed thylakoid membranes and a quite well-structured pyrenoid (Fig. 5j–l). Stromatic starch was visible immersed in the thylakoid system and/or near the pyrenoid (Fig. 5j, l). In some cases, the cell wall had an uneven thickness and appeared to release externally excreted material (Fig. 5j–l). Similar to SA and M cells, but different to autotrophic A ones, some SM cells contained pale globules, ascribable to lipid accumulation (Fig. 5j).

#### Light microscopy characterization

Light microscopy observations were done to improve information on morphology of cells, but also to evaluate some aspects of their biochemical composition; for this purpose, specific staining for EPS and for cytoplasmic lipids was performed (Figs. 6 and 7). Algae A were mostly occupied by a large chloroplast, each containing one pyrenoid (as also evidenced by TEM analyses) (Fig. 6a, b); the organelle was typically green, when conventional white light was used for observations, and red due to the autofluorescence of the chlorophyll, when 436-nm excitation light was used (Fig. 6d). Nile Red staining did not reveal the presence of lipid globules; particularly, stained cells excited at 485 nm did not show the typical gold-yellowish fluorescence due to lipids, but only background autofluorescence of the chlorophyll was detectable (Fig. 6e). Staining with Alcian Blue dye for EPS resulted in a positive reaction at the cell wall level, which appeared slightly and evenly light-blue (Fig. 6c, f). As regards starved autotrophic SA cells, some large and vacuolated cells were visible next to the 2.5–3 μm diameter cells (see above) (Fig. 6g). All cells, similar to algae A, emitted red autofluorescence due to chlorophyll (Fig. 6j). Different to A *N. oleoabundans* cells, SA ones showed a positive reaction to Nile Red staining; in particular, some cells, which appeared granulated under conventional light, emitted the gold-yellow fluorescence linked to lipids, while other cells did not (Fig. 6h, k). In these unstained cells, the granulations were likely due to the stromatic starch granules seen with TEM (Fig. 5e). In SA algae samples, the cell wall was strongly positive to Alcian Blue staining and some light-blue material was released from the cells (Fig. 6i, l).

As regards M and SM samples, they were characterized by cells containing a large, green chloroplast, which emitted red autofluorescence (Fig. 7a, d, g, j). Furthermore, both algal samples presented poorly granulated cells containing lipid droplets, as confirmed by Nile Red staining (Fig. 7b, e, h, k). Mixotrophic M cells positively reacted to Alcian Blue dye, and their cell walls appeared slightly and uniformly stained; however, the most intense staining, involved abundant material released from the cells and sometimes still close to the cell walls, creating a dense agglomerate of algae (Fig. 7c, f). Thus, the strong coloration indicated for an important release of EPS material from M cells. Differently, algae from SM samples, despite the evident reaction to Alcian Blue at the cell wall level, did not release as much light-blue material as the M algae did (Fig. 7i, l).

### Algae extract properties

#### Cells viability following treatment with *N. oleoabundans* algae extracts in N9 microglial cells

**Fig. 8:**
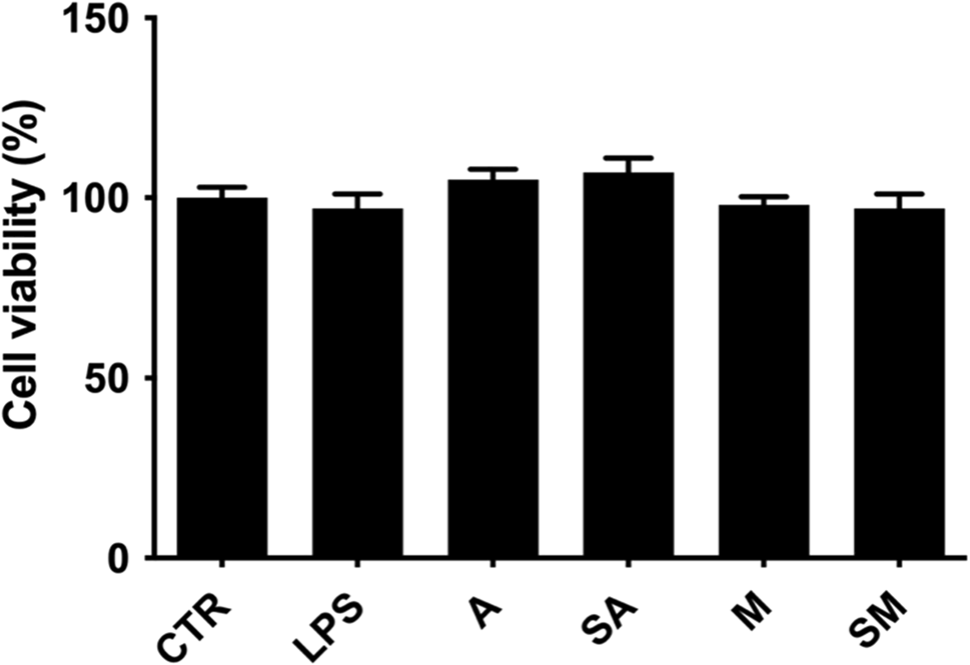
Effect of algae extracts on N9 microglial cell viability. N9 cells were treated with LPS 1 μg mL^−1^ or algae extracts A, SA, M, and SM 1:5 diluted for 24 h, and then, the cell vitality was assessed by MTS assay. Data were expressed as means ± s.d (*n* = 3). Statistical analysis was performed using the one-way ANOVA test followed by Dunnett’s post hoc analysis (*p* < 0.05)

The effects of LPS and algae extracts on cell viability were investigated in N9 microglial cells, chosen as a model of cells involved in inflammation of the central nervous system. In particular, the potential toxicity of LPS and algae extracts was evaluated by using MTS assay. Our results showed that LPS 1 μg mL^−1^ as well as algae samples (A, SA, M, and SM) at 1:1 and 1:5 dilutions did not affect the cell viability of N9 cells for 24 h (Fig. 8). Therefore, the effects of 1:5 dilutions were evaluated in the subsequent experiments.

#### Antioxidant properties of *N. oleoabundans* algae extracts

**Fig. 9:**
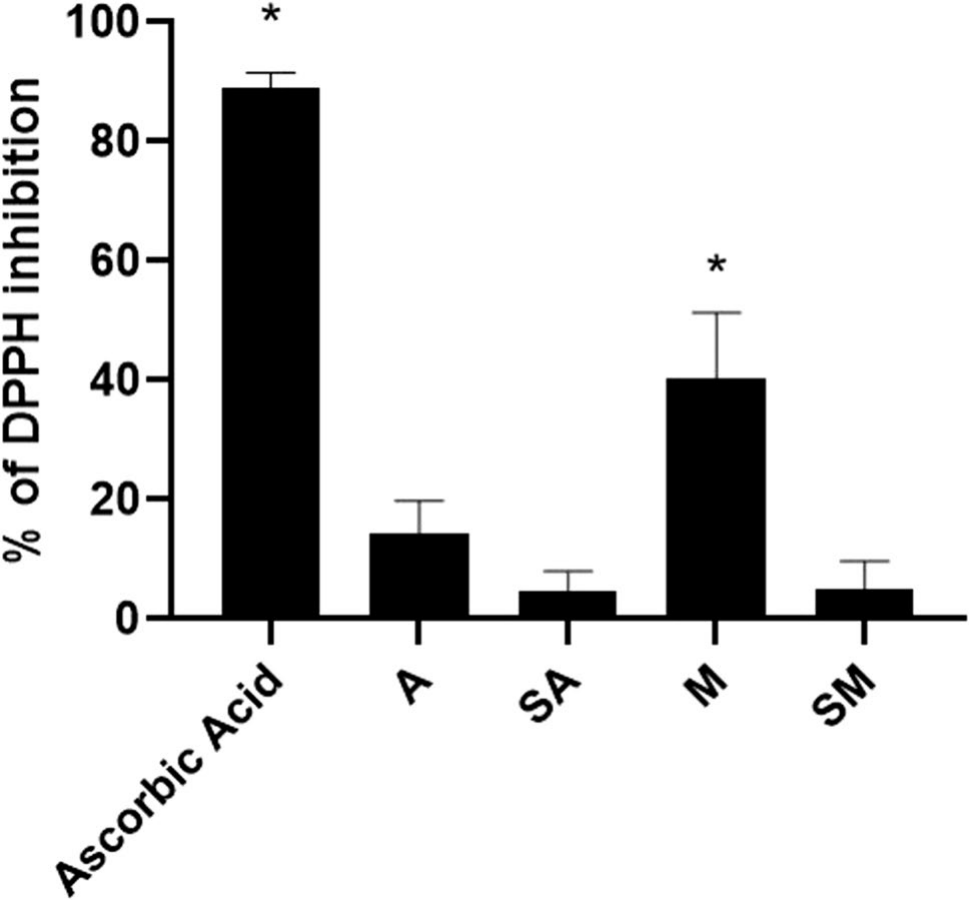
Antioxidant effect of algae extracts on DPPH inhibition. The antioxidant activity of 1:5-diluted algae extracts A, SA, M, and SM and of ascorbic acid 50 μM was evaluated with DPPH 0.1 mM. Data were expressed as mean ± s.d; *p < 0.001 versus control (DPPH alone) (*n* = 3). Statistical analysis was performed using the one-way ANOVA test followed by Dunnett’s post hoc analysis (*p* < 0.05)

Antioxidants are useful because they scavenge the free radicals that induce oxidative stress and inflammation. Providing electron or hydrogen atom, radical scavengers convert the DPPH-free radical into its reduced product (DPPH-H), which has a yellowish color. Therefore, algae extracts were investigated for their antioxidant properties by the DPPH assay. In detail, the results of 1:5-diluted algae extracts, expressed as % of inhibition of DPPH activity, are presented in Fig. 9.

The algae extracts A, SA, and SM did not reduce the radical DPPH activity in a statistically significant way, showing 13 ± 3, 4 ± 2, and 4 ± 3% of inhibition, respectively, while interestingly the M fraction showed a 36 ± 6% of DPPH scavenging activity. The antioxidant ascorbic acid 50 μM was used as an internal positive control in each experiment and was able to strongly reduce DPPH absorbance by 89 ± 1%.

#### Anti-inflammatory properties of algae extracts in N9 microglial cells

**Fig. 10:**
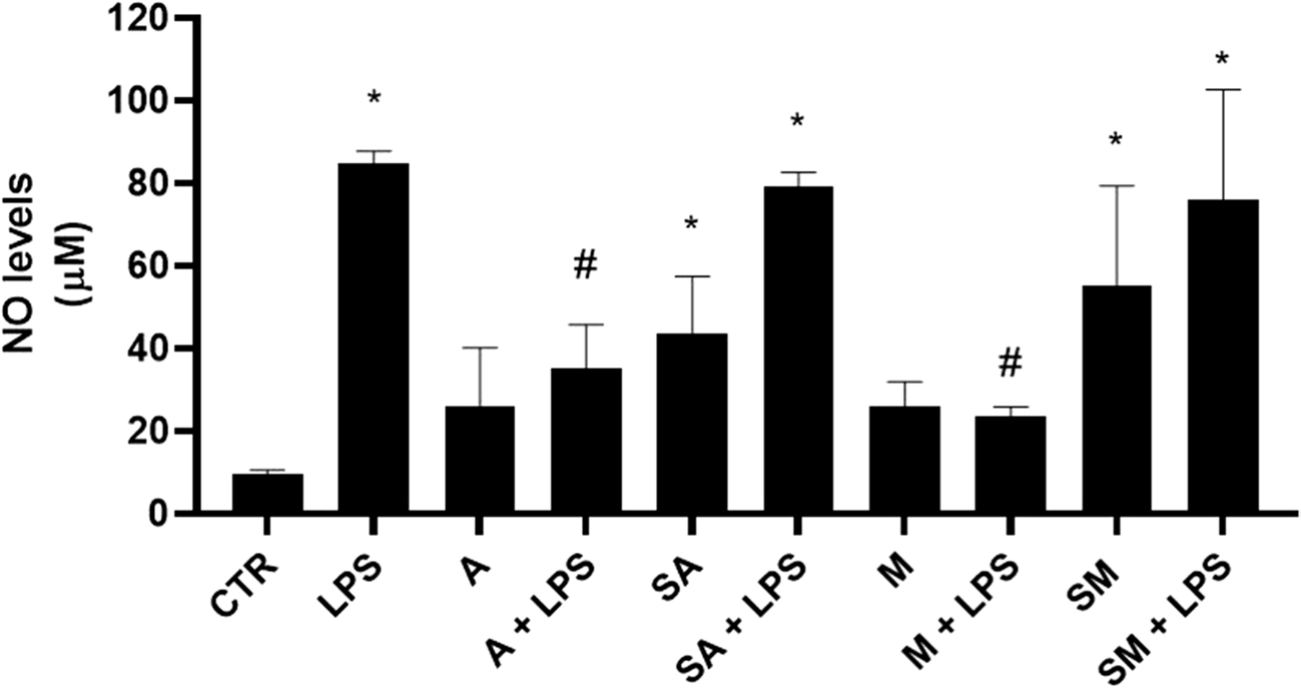
NO inhibition by algae extracts. Effect of 1:5-diluted algae extracts and LPS 1 μg mL^−^^1^ alone or in combination on the NO levels in N9 microglial cells. Data are expressed as means ± s.d; *p < 0.001 versus control; #*p* < 0.001 versus LPS. Statistical analysis was performed using the one-way ANOVA test followed by Dunnett’s post hoc analysis (*p* < 0.05)

The anti-inflammatory properties of algae extracts were tested in N9 microglial cells by nitric oxide (NO) assay. As shown in Fig. 10, extracts from A and M samples alone did not significantly affect the NO production by N9 microglial cells, while SA and SM significantly increased it. As expected, 1 μg mL^−1^ LPS treatment of the cells for 24 h increased NO secretion, reaching a concentration of 85 ± 9 μM. Interestingly, A and M algae extracts significantly reduced NO levels in LPS-activated microglial cells (35 ± 10 μM and 24 ± 2 μM, respectively). This anti-inflammatory effect was not observed with SA and SM algal extracts in combination with LPS (79 ± 6 and 76 ± 27 μM, respectively).

## Discussion

In this work, an integrated approach, including morphological and biochemical techniques, was used to characterize the biology and composition of *N. oleoabundans* cells under different culture conditions; it is indeed well established that different culture conditions of microalgae interfere with their biochemical composition, which in turn can be related to variations in morphology (Azaman et al. 2017; Baldisserotto et al. 2016, 2021, 2023a; da Silva et al. 2023; Neofotis et al. 2021; Shibzukhova et al. 2023; Lu et al. 2022; Yun et al. 2021). To highlight bioactivity, aqueous extracts of all *N. oleoabundans* samples were then tested for their antioxidant and anti-inflammatory activity. It is reported that for complex matrices, as total aqueous extracts are, making a correlation between antioxidant/anti-inflammatory potential and classes of molecules may be ineffective if complete biochemical characterization of the algal biomass is not available (Almendinger et al. 2021). Consequently, as this research paper does not pretend to provide a punctual and full characterization of the entire biochemical profile of the algae under investigation, in order to identify the best cultivation mode for obtaining algal biomass with effective bioactivity, our main intention was aimed at understanding how different cultivation conditions may affect the overall status of *N. oleoabundans*, and then how much the biological response of the alga impacts the antioxidant/anti-inflammatory activity of its extracts.

First of all, this work compared the cultivation of the alga in autotrophic, mixotrophic, and starvation conditions in two-stage cultivation protocols, in which a good yield of algal biomass was supposed to be reached at the end of the first stage (in this work set up under autotrophy or under glucose-induced mixotrophy), while the following stage consisted in cell starvation in tap water as an adverse condition enhancing the synthesis of high-value compounds (Aziz et al. 2020; Ding et al. 2019; Lu et al. 2022). The use of glucose as organic carbon to promote the mixotrophic metabolism in *N. oleoabundans* was chosen because previous studies demonstrated that the alga prefers glucose to other organic carbon sources (sucrose, fructose, or acetate) (Giovanardi et al. 2013). It is indeed well-known that the organic carbon metabolism is species-dependent, so that the use of different organic carbon substrates leads to different growth results (Pang et al. 2019; Baldisserotto et al. 2021; Gulk et al. 2023). As observed in a previous work with *N. oleoabundans* in the same conditions (i.e., same culture medium and organic carbon source and dose) (Baldisserotto et al. 2023a), mixotrophic cultures needed only 12 days of cultivation to reach a cell density 4 times higher than that of autotrophic ones at the same experimental time and maintained quite stable F_*V*_/F_*M*_ values over 0.7 (Fig. 2). Even if direct comparison of the cell density of algae belonging to different species or cultivated under different culture conditions is not possible, since all algae species have specific growth characteristics, the finding of a 4 times higher cell density (and a substantially 3 times higher biomass; Fig. 3a, b) in mixotrophic cultures of *Neochloris* than in autotrophic ones is promising and comparable to that observed for other microalgae cultivated under mixotrophy, for example, *Chlorella* or *Dunaliella*, respectively cultivated with various concentrations of glucose (5 to 25 gL^−1^; 5 to 7 times higher depending on the dose) or 100 mM acetate (3 times higher) (Chavoshi and Shariati 2019; Castillo et al. 2021; Yun et al. 2021). After stage one of cultivation under autotrophy or mixotrophy, starvation interrupted growth and induced a decrease in the photosynthetic overall performance to values between 0.54 and 0.59 (Fig. 2), supporting that starved algae were stressed but still alive. Indeed, in green microalgae, depending on species, F_*V*_/F_*M*_ values of healthy and growing cultures are usually assessed around 0.62–0.65, with lower values in old cultures or in case of stress, and very low values (even below 0.2) in cultures undergoing the death phase (White et al. 2011; Baldisserotto et al. 2014; Goiris et al., 2015; Machado and Soares 2022). Considering that algae in all cultivations were viable, so able to undergo a metabolism (normal or even altered), the best solution for a biotechnological application of the alga in terms of achieved cell density and dry biomass yield was the mixotrophic one.

The addition of glucose to the culture medium boosted *N. oleoabundans* growth (higher cell density and biomass production, and shorter cultivation period compared to autotrophy without glucose; Figs. 2 and 3) and increased its PSII maximum quantum yield, used as a quick and robust index of photosynthetic efficiency and cell vitality (Kalaji et al. 2014; Schagerl et al. 2022), thus supporting previous results on the same alga in the same culture condition (BG11 medium added with 2.5 g L^−1^ of glucose), or also in a brackish medium added with glucose or an organic carbon source, deriving from fruit processing (4- to 16-fold higher cell density—F_*V*_/F_*M*_ values over 0.7; Giovanardi et al. 2013; Baldisserotto et al. 2014, 2016, 2023a). It was reported that the alga, especially when cultivated with glucose, keeps PSII more open than in autotrophy, indicating that the respiration of glucose taken up from the medium feeds the carbon fixation reactions with CO_2_ (Ferroni et al. 2018). Thus, higher PSII maximum quantum yield in glucose-mediated mixotrophic cultures reflects an improved light energy use for a better growth of the alga (Ferroni et al. 2018). One can assume that the metabolism of *N. oleoabundans* can also be modified to enable the alga to a better interaction with the external environment, for example, through the production of protective compounds, like EPS (Figs. 5 and 7), whose production clearly requires energy, besides a carbon source (Wolfaardt et al. 1999). Furthermore, as expected from two-stage cultivation protocols, resuspending algae in water resulted in a state of stress: no further growth was observed, and F_*V*_/F_*M*_ values clearly decreased as a sign of photoinhibition, especially towards the end of the starvation period (Fig. 2). The decrease in F_*V*_/F_*M*_, which is linked to the production of harmful reactive oxygen species in chloroplasts (Murata et al. 2007; Endo et al. 2023), was more evident in starved autotrophic cultures than in the mixotrophic starved ones, analogous to what reported in a previous work on two-stages cultivation of *N. oleoabundans* in a brackish medium (Baldisserotto et al. 2014). In that case, starved autotrophic cultures, starting from values of F_*V*_/F_*M*_ of about 0.65, let quickly record values around 0.4, while starved mixotrophic ones underwent a less marked decrease, from 0.77 to 0.6. Overall, as compared to M samples, photoinhibition highly affected both starved SA and SM cultures, but also, even if at lower level, the autotrophic ones.

Stated that evident stress occurred in SA and SM samples and that the most photo-protected samples were the mixotrophic ones, a biochemical and morphological characterization of all samples was done to highlight possible effects on overall content in antioxidant and anti-inflammatory compounds in the alga. Interestingly, parallel to higher F_*V*_/F_*M*_ values, M samples showed high appression of the thylakoid membranes (Fig. 5g–i), which can be justified by a strong association of light harvesting complex II (LHCII) to PSII (Giovanardi et al. 2017). Thus, TEM observations revealed a strong assembly of thylakoid membranes with a proper association of photosynthetic pigments and proteins. Mixotrophic algae were characterized by a relatively low content of photosynthetic pigments compared to autotrophic algae but showed a Chl*a*/Chl*b* molar ratio similar to that of autotrophic cells, pointing to a good stoichiometry of pigments for light harvesting and use, and high chlorophylls over carotenoids ratio, due to low levels of carotenoids rather than low chlorophyll content (Fig. 4c–f). This finding, together with fluorimetric analysis, suggests a better use of light, mainly due to chlorophylls, rather than extensive production of carotenoids, which, on the other side, protect in general against oxidative stress (Vaquero et al. 2014; Gong and Bassi 2016; Novevoska et al. 2019). The concomitant finding that mixotrophic extracts have a remarkable antioxidant potential, as revealed by the DPPH test (Fig. 9), implicates that carotenoids are not the main players in the anti-oxidative properties of the alga when cultivated in the presence of glucose. In the present experimentation, the percentage of DPPH inhibition was around 40% or slightly below 20% for M and A extracts, respectively (Fig. 9). Results for A samples are in line with those reported by Banskota et al. (2019) for the same alga but cultivated under continuous illumination in f/2 low salinity medium (15%), while M extracts showed as high DPPH scavenging activity as that recorded for the most promising algae tested by the researcher, *Tetraselmis chui* and *Porphyridium aeruginosa* (45 and 38%). In that study, for *N. oleoabundans*, the low antioxidant property was linked to low content in phenolics (26.6 μmol GAE g^−1^ DW) (Banskota et al. 2019). Such values were quite similar to those found for autotrophic *N. oleoabundans* in the present work (25 mg_Eq_ g^−1^ DW; Fig. 4b); even lower values found in mixotrophic algae point to the involvement of other compounds to support the higher antioxidant property here recorded (40% of DPPH inhibition; Fig. 9).

Mixotrophic algae also reacted to the addition of glucose into the culture medium by producing intracellular lipids and EPS, the latter being mainly extruded from the cell wall (Figs. 5 and 7). Lipids, which are often overproduced in mixotrophic algae as a response to a fast consumption of nutrients in the medium, thus inducing a stress condition similar to starvation (Baldisserotto et al. 2016, 2021; Huang et al. 2021; Paranjape et al. 2016), are involved in antioxidant and anti-inflammatory responses (Volkman 2016; Sansone and Brunet 2019; Galasso et al. 2019). On the other hand, EPS are polymeric substances often produced by microalgae to deal with changes in environmental conditions, organic carbon addition included, and are considered as promising antioxidant, immunomodulatory bioactive agents (Li et al. 2020; Babiak and Krzemìnska 2021; Zhou et al. 2023). The production of EPS requires both energy and carbon sources, which are theoretically well-available in mixotrophic conditions (Wolfaardt et al. 1999). Li et al. (2020) isolated and characterized an EPS fraction from mixotrophic *N. oleoabundans* cultivated in the presence of 10 g L^−1^ glucose; they found that the dominant fraction was a proteoglycan (molecular weight, 5.17 × 10^5^ Da), containing 0.59% (w/w) of peptide, and glucose, mannose, galactose, xylose, ribose, arabinose, and rhamnose as the polysaccharidic part, glucose, mannose, and galactose being the main components. The same authors found that only glucose (among sugars they have tested, i.e., glucose, mannose, lactose, galactose, and sucrose) promoted the production of a bioactive EPS fraction, thus pointing to the need to better characterize also the EPS produced by *N. oleoabundans* in our study. A more detailed investigation in this regard is also justified by the importance of other aspects related to the chemistry of EPS. Indeed, glucuronic acid and sulfate residues, often present in the backbone of polysaccharides, appear crucial for biomedical purposes, such as against inflammation (Xiao and Zheng 2016; de Jesus Raposo et al. 2015). Similar to our work, Li et al. (2020) did not evaluate sulfation levels of their EPS fraction from *N. oleoabundans*, however a good variety of green microalgae (*C. stigmatophora*, *C. autotrophic*a, *C. vulgaris*, *Tetraselmis tetrathele*, *Dunaliella tertiolecta*, and *D. salina*) has been found to have EPS with high levels of sulfation (Amaro et al. 2011; Guzman-Murillo and Ascencio 2000; Ogawa et al. 1997, 1999; de Jesus Raposo et al. 2015; Xiao and Zheng 2016), thus supporting the idea that also the exopolysaccharides produced by *N. oleoabundans* can have a similar peculiarity; verifying this could strengthen the mechanism underlying the anti-inflammatory response observed in microglial cells in this work. Differently, the similar anti-inflammatory effects recorded for extracts of autotrophic algae could be more likely linked to their higher content in both photosynthetic pigments, polyphenolics, and proteins rather than in EPS or lipids since the former were higher in A than in M samples, and, vice versa, EPS and lipids in M than in A algae (Figs. 4, 5, 6, 7 and 9). Similar result was also obtained in a previous work with the same alga in both autotrophic and mixotrophic conditions, suggesting a robust response of the alga to variation in cultivation mode (Baldisserotto et al. 2023a). The overall inverted composition in those samples resulted in a synergistic anti-inflammatory effect; all the detected compounds are known to have bioactivity against inflammation (Coulombier et al. 2021).

For starved algae, both after autotrophic and mixotrophic cultivation during the first phase of trials, no interesting responses in terms of antioxidant and anti-inflammatory activity of the derived extracts was recorded. In both cases, the low metabolic activity due to nutrient deficiency was reflected in the block of the algal growth and in a lower content, with respect to A samples, in protein, pigment, and phenolics content (Fig. 4), with an apparent impact on both antioxidant and anti-inflammatory capacities of their aqueous extracts. Lipids highlighted for starved algae (Fig. 5) appeared to be not sufficient to guarantee noteworthy antioxidant and anti-inflammatory properties (Figs. 9 and 10).

Reduction in protein and pigments content in response to nutrient stress condition is not surprising, due to insufficient availability of N and P for their synthesis (Liang et al. 2013; Goiris et al., 2015; da Silva Ferreira and Sant’Anna 2017), while the decrease in phenolics is less obvious. In microalgae the biological meaning of these compounds is to counteract adverse environmental conditions, due to several abiotic factors, thus suggesting that they should increase under stress conditions (Gauthier et al. 2020; Kapoore et al. 2021; Cichónski and Chrzanowski 2022; Faraloni et al. 2021). Thus, considering that in our tests, starvation caused a decrease in the photosynthetic efficiency, a condition which usually promotes oxidative stress and accumulation of excess energy, with consequent induction of photo-protective mechanisms, including synthesis of antioxidants (Faraloni et al. 2021), an increase in phenolics could have been assumed. Nevertheless, for our starved *N. oleoabundans* cells, we observed a strong decrease in these antioxidant compounds. A similar decrease in this class of molecules was observed for the first time by Goiris and coworkers (2012) in 3 microalgae species, two chlorophytes and a diatom, cultivated under nutrient deficiencies (about twofold less total phenolics in *C. vulgaris* and *Phaeodactylum tricornutum* exposed to N and P limitation; similar phenolics content in *Tetraselmis suecica* under control and P-limited conditions, and a significantly lower content in samples under N limitation). Similarly, synthesis of cell wall EPS was induced in starved algae as a response to harsh culture conditions (Babiak and Krzemìnska 2021), but they mainly contributed to cell wall enlargement (especially in SA samples; Fig. 5d–f), rather than being released outside the cells, showing a response of *N. oleoabundans* different from that observed when glucose was added to the culture medium.

Overall, these results confirm the strict need for thoroughly studying the biology of each microalgal species under varying culture conditions, especially if the alga is meant for a specific biotechnological purpose. Changing the way microalgae are grown, in fact, may not yield such obvious results.

## Conclusions

Overall *N. oleoabundans* is a good candidate for the production of target bioactive products with antioxidant/anti-inflammatory properties in a combination suitable to obtain promising extracts for biotechnological applications. Tentatively, one would be inclined to infer that the *N. oleoabundans* with the highest antioxidant and anti-inflammatory properties may be that cultivated autotrophically, in which the whole morpho-physiology of the alga is optimal for an efficient algal growth. However, it has been observed that the most promising growing condition to produce an antioxidant algal biomass with anti-inflammatory properties is the mixotrophic one, which promotes in the alga an overall stronger photosynthetic metabolism with characteristics of higher photo-protection from oxidative damage, probably due also to higher production of EPS and lipids. Thus, the mixotrophic alga reacts efficiently to the environment, and its response is reflected in greater overall antioxidant and anti-inflammatory power of its aqueous extract. Furthermore, mixotrophy appears preferable for obtaining optimal algal biomass to produce natural bioactive extracts that are potentially well tolerated by human metabolism (with fewer side effects than synthetic products) and environmentally sustainable, considering, for example, the positive effect that microalgae cultivation can have from a carbon sequestration perspective (Kornienko et al. 2019; Sadvakasova et al. 2023).

The use of a microglia cell line in this work to test the anti-inflammatory effect of the four algal extracts represents a first finding related to the potential of mixotrophic *N. oleoabundans* to alleviate inflammation in neurodegenerative diseases, a scourge that affects the world’s population more and more extensively. Nevertheless, it cannot be excluded that the different peculiarities of the algae in different modes of cultivation may also play a role towards other issues related to the health and well-being not only of humans, but also of animals, up to that of crops (Bello et al. 2021; Mavrommatis et al. 2023).

## Supplementary Information

Below is the link to the electronic supplementary material.

Supplementary file1 (DOCX 17 KB)

Supplementary file2 (DOCX 16 KB)

## Data Availability

The datasets generated during the current study are available from the corresponding author on reasonable request.
