# Supplementary material for: Cultivation modes affect the morphology, biochemical composition, and antioxidant and anti-inflammatory properties of the green microalga Neochloris oleoabundans
Source: Protoplasma. 2024 Jun 12;261(6):1185–206. doi: 10.1007/s00709-024-01958-7 (PMC11511745; doi:10.1007/s00709-024-01958-7)
Supplement: Supplementary file 1 — Supplementary file1 (DOCX 17 KB) [file 709_2024_1958_MOESM1_ESM.docx]

**Article Title:** Cultivation modes affect the morphology, biochemical composition, and antioxidant and anti-inflammatory properties of the green microalga *Neochloris oleoabundans*

**Journal Title:** Protoplasma

**Authors’ names:** Baldisserotto C, Gessi S, Ferraretto E, Merighi S, Ardondi L, Giacò P, Ferroni L, Nigro M, Travagli A, Pancaldi S

**Corresponding author:** Prof. Simonetta Pancaldi; affiliation: Department of Environmental and Prevention Sciences, University of Ferrara, C.so Ercole I d’Este, 32, 44121 Ferrara – Italy; e-mail address: simonetta.pancaldi@unife.it

**Table S1.**

Recipe of BG11 synthetic mineral medium (from https://utex.org/products/bg-11-medium).

| **Product** | **Quantity in 1 L** | **Final concentration** |
| --- | --- | --- |
| NaNO_3_ | 1.5 g | 17.6 mM |
| K_2_HPO_4_ | 0.04 g | 0.23 mM |
| MgSO_4_^.^7H_2_O | 0.075 g | 0.3 mM |
| CaCl_2_^.^2H_2_O | 0.036 g | 0.24 mM |
| Citric acid | 0.006 g | 0.031 mM |
| Ammonium ferric citrate | 0.006 g | 0.021 mM |
| EDTA(Na_2_) | 0.001 g | 0.0027 mM |
| Na_2_CO_3_ | 0.02 g | 0.19 mM |
| Trace metal solution | 1 mL |  |
| H_2_O | To 1 L |  |

**Trace metal solution**:

| **Product** | **Quantity in 1 L** | **Final concentration** |
| --- | --- | --- |
| H_3_BO_3_ | 2.86 g | 46 mM |
| MnCl_2_^.^4H_2_O | 1.81 g | 9 mM |
| ZnSO_4_^.^7H_2_O | 0.222 g | 0.77 mM |
| Na_2_MoO_4_^.^2H_2_O | 0.39 g | 1.6 mM |
| CuSO_4_^.^5H_2_O | 0.079 g | 0.3 mM |
| Co(NO_3_)2^.^6H_2_O | 49.4 mg | 0.17 mM |
| H_2_O | To 1 L |  |
