# Supplementary material for: Cultivation modes affect the morphology, biochemical composition, and antioxidant and anti-inflammatory properties of the green microalga Neochloris oleoabundans
Source: Protoplasma. 2024 Jun 12;261(6):1185–206. doi: 10.1007/s00709-024-01958-7 (PMC11511745; doi:10.1007/s00709-024-01958-7)
Supplement: Supplementary file 2 — Supplementary file2 (DOCX 16 KB) [file 709_2024_1958_MOESM2_ESM.docx]

**Article Title:** Cultivation modes affect the morphology, biochemical composition, and antioxidant and anti-inflammatory properties of the green microalga *Neochloris oleoabundans*

**Journal Title:** Protoplasma

**Authors’ names:** Baldisserotto C, Gessi S, Ferraretto E, Merighi S, Ardondi L, Giacò P, Ferroni L, Nigro M, Travagli A, Pancaldi S

**Corresponding author:** Prof. Simonetta Pancaldi; affiliation: Department of Environmental and Prevention Sciences, University of Ferrara, C.so Ercole I d’Este, 32, 44121 Ferrara – Italy; e-mail address: simonetta.pancaldi@unife.it

**Table S2.**

Composition of tap water used for experimentations (from https://www.gruppohera.it/offerte-e-servizi/casa/acqua/che-acqua-bevi/archivio; considered period: second semester 2022).

| **Parameter** | **Value** |
| --- | --- |
| Bicarbonate alkalinity | 197 mg L^-1^ |
| Total alkalinity | 164 mg L^-1^ |
| NH_4_^+^ | < 0.02 mg L^-1^ |
| As | 2 mg L^-1^ |
| Ca | 54 mg L^-1^ |
| Cl (free residual) | 0.2 mg L^-1^ |
| Cl^-^ ion | 35 mg L^-1^ |
| pH | 7.7 |
| Conductivity | 513 µS/cm at 20°C |
| Hardness | 19°F |
| Fl^-^ ion | < 10 mg L^-1^ |
| Mg | 13 mg L^-1^ |
| Mn | < 5 mg L^-1^ |
| Nitrate (NO_3_^-^) | 7 mg L^-1^ |
| Nitrite (NO2^-^) | < 0.02 mg L^-1^ |
| K | 3 mg L^-1^ |
| Na | 20 mg L^-1^ |
| Sulphate (SO_4_^3-^) | 42 mg L^-1^ |
| Dry residue at 180°C | 265 mg L^-1^ |
